# Ultralow-dose binary oncolytic/helper-dependent adenovirus promotes antitumor activity in preclinical and clinical studies

**DOI:** 10.1126/sciadv.ade6790

**Published:** 2023-03-29

**Authors:** Daniel Wang, Caroline E. Porter, Bora Lim, Amanda Rosewell Shaw, Catherine S. Robertson, Mae L. Woods, Ya Xu, Greyson G.W. Biegert, Daisuke Morita, Tao Wang, Bambi J. Grilley, Helen Heslop, Malcolm K. Brenner, Masataka Suzuki

**Affiliations:** ^1^ Department of Medicine, Baylor College of Medicine, Houston, TX, USA.; ^2^ Center for Cell and Gene Therapy, Baylor College of Medicine, Texas Children’s Hospital, Houston Methodist Hospital, Houston, TX, USA.; ^3^ Duncan Cancer Center-Breast, Baylor College of Medicine, Houston, TX, USA.; ^4^ Department of Pathology and Immunology, Baylor College of Medicine, Houston, TX, USA.; ^5^ Dan L Duncan Comprehensive Cancer Center, Baylor College of Medicine, Houston, TX, USA.; ^6^ Department of Pediatrics, Baylor College of Medicine, Houston, TX, USA.

## Abstract

We show that a binary oncolytic/helper-dependent adenovirus (CAdVEC) that both lyses tumor cells and locally expresses the proinflammatory cytokine IL-12 and PD-L1 blocking antibody has potent antitumor activity in humanized mouse models. On the basis of these preclinical studies, we treated four patients with a single intratumoral injection of an ultralow dose of CAdVEC (NCT03740256), representing a dose of oncolytic adenovirus more than 100-fold lower than used in previous trials. While CAdVEC caused no significant toxicities, it repolarized the tumor microenvironment with increased infiltration of CD8 T cells. A single administration of CAdVEC was associated with both locoregional and abscopal effects on metastases and, in combination with systemic administration of immune checkpoint antibodies, induced sustained antitumor responses, including one complete and two partial responses. Hence, in both preclinical and clinical studies, CAdVEC is safe and even at extremely low doses is sufficiently potent to induce significant tumor control through oncolysis and immune repolarization.

## Teaser

Intratumoral treatment with CAdVEC activates host immune responses against cancer cells in both pre-clinical and clinical studies.

## INTRODUCTION

Oncolytic viruses (OVs) selectively replicate in and kill tumor cells and can induce immune cell infiltration at tumor sites through virus-mediated inflammatory responses (*1*, *2*) that may reduce the immuno-inhibitory effects of the preexisting tumor microenvironment (TME) (*2*). To further enhance host antitumor immunity through OVs, investigators have incorporated immunomodulatory molecules into their OVs (“Armed” OVs), which demonstrated additive antitumor effects in preclinical and clinical studies (*3*). However, modulation of a single immune pathway is insufficient to abrogate immune resistance to OVs due to the multilayered defenses of solid tumors against immune attack (*4*). We therefore developed a binary oncolytic/helper-dependent adenovirus (Ad) system (CAdVEC), coinfecting oncolytic Ad (OAd) with helper-dependent Ad (HDAd), which has a cargo capacity of up to 34 kb and is able to express multiple immunomodulatory molecules in a single vector (*5*). We previously demonstrated that CAdVEC expressing PD-L1 blocking antibody enhances tumor-infiltrating T cell activity (*6*) and additional expression of interleukin-12p70 (IL-12p70) enhances T cell proliferation/expansion (*7*). This dual expressing CAdVEC leads to better tumor control than either of the single expression vector CAdVEC.IL-12 or CAdVEC.PD-L1 (*7*).

The inclusion of an HDAd as a component of CAdVEC not only can simultaneously provide countermeasures to several different tumor inhibitory mechanisms but also may enable more durable antitumor activity. In contrast to expressing immunomodulatory genes directly in an Armed OV, HDAds are nonlytic and have no viral genes in their vector DNA, providing an opportunity for continued expression of genes beneficial to the development of a sustained antitumor immune response. Studies in nonhuman primates, for example, showed that a single injection of HDAd can express transgenes for more than 5 years (*8*). If these benefits extend to immunostimulatory gene expression in TME, then the CAdVEC system should have greater potency than conventional Armed OVs, thereby eliminating the need for injecting doses of virus that are intrinsically toxic (*9*) or technically unfeasible. While the issue of ultimate efficacy can only be validated by prolonged large-scale clinical studies, here, we investigate the potency of CAdVEC in preclinical models and in a pilot clinical study by administering a single dose of CAdVEC that is 100-fold lower than the lowest doses studied in previous Armed OAd trials (*7*–*9*). When combined with an equally low dose of HDAd expressing IL-12p70, anti–PD-L1 antibody, and HSVtk safety switch, this ultralow-dose combination OAd/HDAd therapy modulated local and systemic immune activity, leading to partial and complete tumor responses in both preclinical studies and humans.

## RESULTS

### Local ULCA elicits antitumor effects in breast cancer xenograft models

**Fig. 1.**
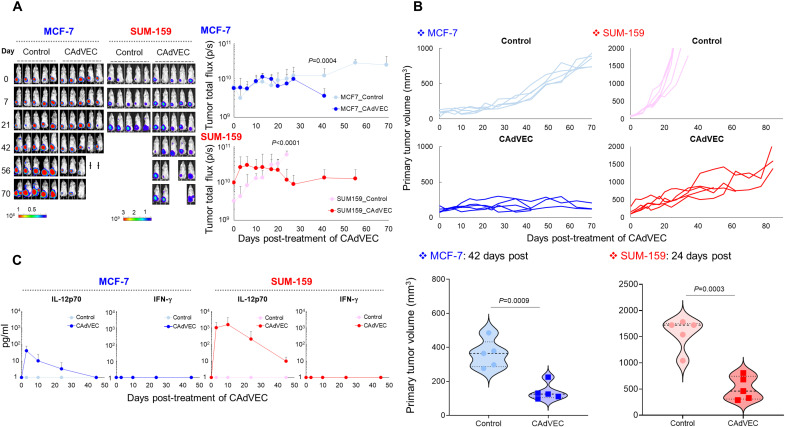
ULCA controls primary tumor growth in breast cancer xenograft mouse models. (**A**) ffLuc-labeled MCF-7 and SUM-159 cells were orthotopically transplanted into the mammary fat pad of NSG female mice (*n* = 5 animals per condition). After tumor volume reached >100 mm^3^, we injected a total of 1 × 10^6^ vp of CAdVEC (OAd:HD = 1:1) intratumorally. Control mice received vehicle (PBS) alone. We monitored tumor bioluminescence at the indicated time points. Data are presented as means ± SD. Two MCF-7 mice treated with CAdVEC were euthanized at 44 days after CAdVEC injection due to coronavirus disease (COVID) animal experiment restrictions. (**B**) Primary tumor volumes were monitored at different time points. Tumor volumes shown here are from 42 days (MCF-7) and 24 days (SUM-159) after injection of CAdVEC; individual data points are represented in violin plot. (**C**) We collected serum samples from mice at 0, 3, 7, 21, and 42 days after injection of CAdVEC and measured IFN-γ and IL-12p70 levels by enzyme-linked immunosorbent assay (ELISA). Data are presented as means ± SD.

We used human xenograft mouse models to evaluate the ability of CAdVEC to directly kill human tumor cells at doses 1000-fold lower than evaluated for other Armed OAds in mouse studies (*10*–*13*). First, we orthotopically transplanted estrogen and progesterone receptor–positive (ER/PR^+^) breast cancer line MCF-7 and triple-negative breast cancer (TNBC) line SUM-159 labeled with firefly luciferase (*ffLuc*) to NSG mice. After tumor volumes reached >100 mm^3^, we injected 1 × 10^6^ total CAdVEC viral particles (vp) intratumorally, representing 0.5 × 10^6^ OAd mixed with 0.5 × 10^6^ HDAd. We then monitored tumor growth (Fig. 1, A and B) and found that a single administration of ultralow-dose CAdVEC (ULCA) significantly controlled primary tumor growth in both models (MCF-7: *P* = 0.0009, SUM-159: *P* = 0.0003). We detected circulating IL-12p70, a gene encoded by the HDAd component of our CAdVEC, in mice that received CAdVEC but not control mice (Fig. 1C). We saw no weight loss or other evidence of toxicity in either animal model (fig. S1). Thus, ULCA significantly controls primary tumor growth in both ER/PR^+^ breast cancer and TNBC xenograft mouse models and produces systemically detectable levels of encoded transgene IL-12p70. In previous studies, we detected CAdVEC-derived PD-L1 antibody at treated tumor sites but not in blood samples (*6*), suggesting that PD-L1 antibody produced by our CAdVEC will not lead to the systemic toxicities seen in patients systemically infused with immune checkpoint inhibitor (ICI) (*14*). To compare the benefits of CAdVEC (combination with OAd and HDAd) with single agents, we evaluated the toxicity, antitumor efficacy, and circulating IL-12p70 levels after low-dose (1 × 10^6^ vp) and high-dose (1 × 10^9^ vp) injection of single agents in the SUM-159 xenograft model (fig. S2). Mice treated with high-dose OAd had significantly more elevated liver enzyme alanine transaminase than mice treated with low-dose OAd (*P* = 0.0248) or HDAd (*P* = 0.0401). Although mice treated with HDAds had levels of IL-12p70 in blood that were proportionate to the dose of CAdVEC, there was no antitumor activity in animals treated with HDAds compared to control mice. While these data indicate that low-dose OAd is sufficient in an immunodeficient setting, our goal is to make an effective product for translation to immunocompetent patients in a clinical setting.

### Local treatment with ULCA controls tumor growth by eliciting host immune responses in breast cancer humanized mouse models

To address whether the immunomodulatory molecules (IL-12p70 and PD-L1 antibody) derived from ULCA recruit the host immune system and further enhance the antitumor effects of the therapy, we evaluated the antitumor activity of CAdVEC in humanized mice (fig. S3) in which human innate and adaptive immune cells are reconstituted (*15*). These mice produce potent responses against human tumors and viruses (*16*).

**Fig. 2.**
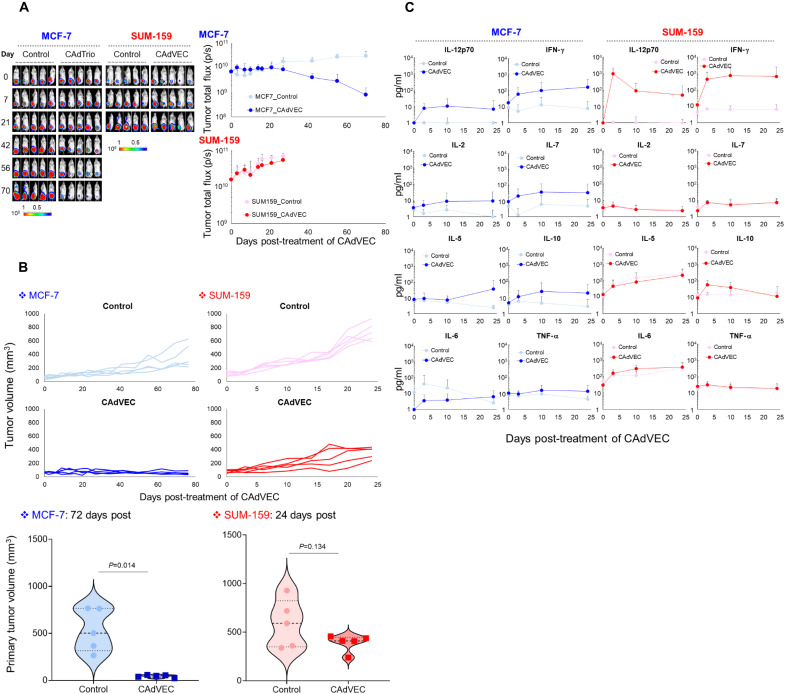
ULCA controls tumor growth in humanized mouse models. (**A**) ffLuc-labeled MCF-7 and SUM-159 cells were orthotopically transplanted into the mammary fat pad of humanized female mice (*n* = 5 animals per condition). After tumor volume reached >100 mm^3^, we intrathecally injected a total of 1 × 10^6^ vp of CAdVEC (OAd:HD = 1:1). Control mice received vehicle (PBS) alone. We monitored tumor bioluminescence at the indicated time points. Data are presented as means ± SD. (**B**) Primary tumor volumes were monitored at different time points. Tumor volumes shown here are from 72 days (MCF-7) and 24 days (SUM-159) after injection of CAdVEC; individual data points are represented in violin plot. (**C**) We collected serum samples from mice at 0, 3, 7, and 21 days after injection of CAdVEC and measured human T_H_1 and T_H_2 cytokine levels by Multiplex. IL-1β was undetectable level. Data are presented as means ± SD.

In both the MCF-7 and SUM-159 humanized mouse models (fig. S3), ULCA (1 × 10^6^ vp) controlled primary tumor growth (Fig. 2, A and B). We measured circulating human cytokine levels in both tumor models and found that CAdVEC treatment induced expression of T helper 1 (T_H_1) [e.g., interferon-γ (IFN-γ)] and T_H_2 cytokines (e.g., IL-10), in addition to transgenic IL-12p70 (Fig. 2C). Local ULCA injection did not, however, increase levels of inflammatory cytokines IL-6 and tumor necrosis factor–α (TNF-α) in either tumor model. These data indicate that ULCA locally activates host immune response but does not induce the pattern of systemic inflammatory cytokines associated with cytokine release syndrome (*17*). In contrast to untreated control mice, which developed metastatic disease, MCF-7 mice treated with ULCA did not develop metastasis. These results are similar to those we saw in our xenograft model. These data indicate that ULCA products directly affected the growth of MCF-7 primary tumor sufficiently to prevent metastasis in xenograft (NSG) mice because there was no immune system in NSG mice. In humanized mice, additional effects on metastasis were indirect and attributed to recruitment of immune cells. Although local CAdVEC treatment did not control SUM-159 tumor metastasis in xenograft mice, it did attenuate metastatic SUM-159 tumor growth in humanized mice, consistent with recruitment of human immune effector cells. We also evaluated the benefits of CAdVEC compared to single agents in SUM-159 humanized mice (fig. S4). In contrast to the marked antitumor effects of low-dose CAdVEC shown in Fig. 2B, each component individually had less antitumor activity. This effect correlated with lower production of proinflammatory cytokines from single agents versus CAdVEC. Although high-dose OAd controlled tumor growth, these mice showed high levels of inflammatory cytokines (e.g., IL-6) compared to low-dose CAdVEC shown in Fig. 2C. These results suggest both oncolysis and immune stimulation are required if tumor control is to be obtained in the absence of profound inflammation. Because OAd-treated patients showed dose-dependent inflammatory responses in other clinical trials (*18*–*20*), these data also suggest that humanized mouse models are appropriate preclinical animal models for OAd-based immunotherapy, including CAdVEC.

### ULCA repolarizes the TME and adaptive immune responses in breast cancer humanized mouse models

To discover whether ULCA also modulated the immuno-inhibitory TME through oncolysis and immunomodulatory molecules in our humanized mouse models (*21*), we repeated the above experiments and harvested tumors 3 weeks after ULCA treatment. We phenotyped tumor-infiltrating immune cells (fig. S5), pooling tumors from four to five humanized mice with MCF-7 tumors, which represents an immunologically “cold” tumor with limited immune cell infiltration, and from SUM-159, which represents an immunologically “hot” tumor with extensive lymphoid infiltrate even in control animals.

**Fig. 3.**
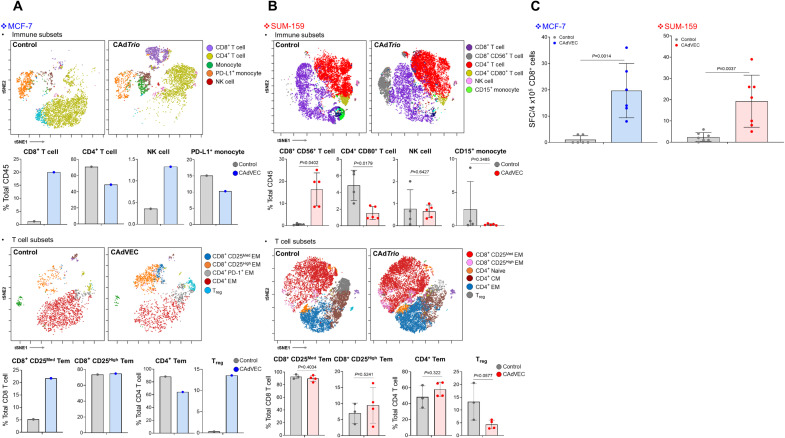
ULCA repolarizes the TME and induces adaptive immune responses against cancer cells in humanized mouse models. MCF-7 and SUM-159 cells were orthotopically transplanted into the mammary fat pad of humanized female mice. After tumor volume reached >100 mm^3^, a total of 1 × 10^6^ vp of CAdVEC (OAd:HD = 1:1) were injected intrathecally. Tumor, spleen, and blood samples were collected at 24 days after CAdVEC injection. (**A**) Because of limited human immune cells in MCF-7 tumors, we pooled immune cells from four to five MCF-7 tumors and stained for flow cytometry and phenotyping. Immune cells from repeated experiment were stained with T cell markers. Single-stained *t*-distributed stochastic neighbor embedding (tSNE) plots are in fig. S5. (**B**) Immune cells from SUM-159 tumors were stained for flow cytometry and phenotyped. Immune cells from repeated experiment were stained with T cell markers. Data are presented as means ± SD. Single-stained tSNE plots are in fig. S5. (**C**) CD8^+^ T cells were isolated from spleen samples, and we performed an IFN-γ ELISPOT assay with irradiated cancer cells (E:T = 10:1). Data are presented as means ± SD.

In MCF-7 tumors, ULCA treatment increased CD8^+^ T cell and natural killer (NK) cell infiltration compared to controls and reduced the number of PD-L1–positive monocytes (Fig. 3A). Moreover, CD8^+^ cells in MCF-7 mice treated with ULCA had increased numbers of activated effector memory T cells compared to control mice. ULCA treatment also increased MCF-7 tumor infiltration of CD4^+^ T cells that had a predominant CD4^+^, CD25^High^, CTLA-4^High^ phenotype, associated with T regulatory (T_reg_) and activated T_H_2 T cells. Hence, ULCA induces a diverse human T cell response in humanized mice.

In contrast to MCF-7 tumors, even untreated SUM-159 tumors had both CD4^+^ and CD8^+^ T cell infiltration (Fig. 3B). Nonetheless, ULCA treatment further increased the number of activated (CD56^+^) CD8^+^ T cells compared to controls (*P* = 0.0402). CD15^+^ monocytes, associated with immunosuppressive monocytes (*22*, *23*), were also reduced in ULCA-treated compared to control mice (*P* = 0.3485). Thus, in SUM-159 mice, ULCA enhanced infiltration of proinflammatory and cytotoxic T cells and reduced the presence of cells with a T_reg_ (CD4^+^, CD25^High^, CTLA-4^High^) phenotype (*P* = 0.108).

We next examined how ULCA-dependent TME repolarization contributes to the development of adaptive immune responses in these models (Fig. 3C and fig. S6). We isolated CD8^+^ T cells from the spleen of these mice and performed IFN-γ enzyme-linked immunospot (ELISPOT) assay with irradiated cancer cells (*24*). We found that ULCA treatment significantly increased cancer cell–reactive CD8^+^ T cell spot numbers compared to control mice in both models (MCF-7; *P* = 0.0014, SUM-159; *P* = 0.0037). Thus, ULCA treatment repolarizes the immunosuppressive TME toward an immune active phenotype in both cold and hot tumors.

### Local ULCA injection is safe in patients

**Fig. 4.**
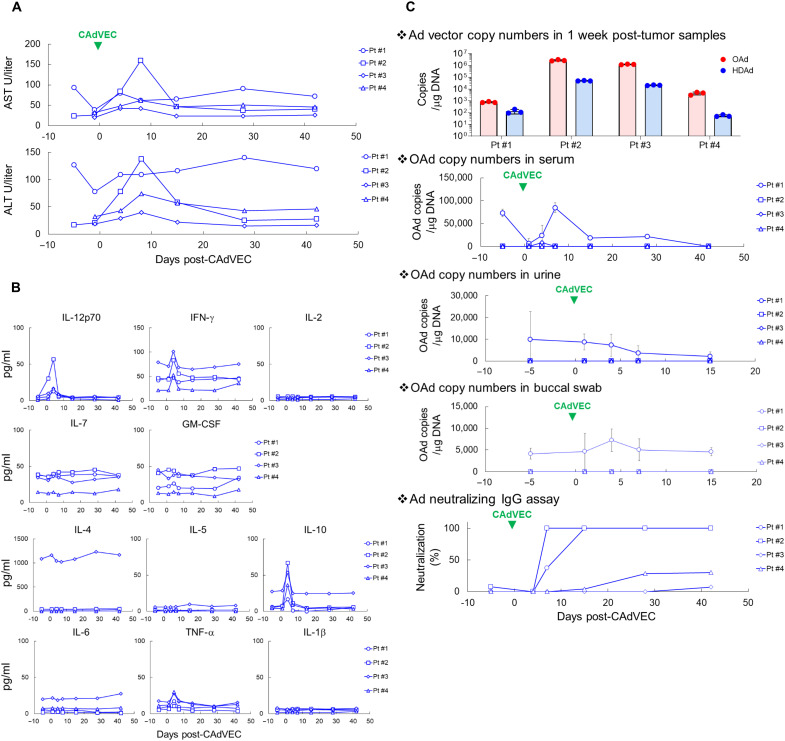
ULCA treatment is safe in patients. A total of 5 × 10^9^ vp of CAdVEC (OAd:HD = 1:1) in 500 μl were intratumorally injected. (**A**) We measured liver enzymes aspartate aminotransferase (AST) and alanine transaminase (ALT) at the indicated time points. (**B**) We measured human T_H_1 and T_H_2 cytokine levels in patient plasma samples at the indicated time points by Multiplex. (**C**) We extracted DNA from 1 week after CAdVEC biopsy samples and determined the copy number of each Ad vector by quantitative polymerase chain reaction (PCR). Serum, urine, and buccal swab were collected at the indicated time points. DNA was extracted from these samples, and the copy number of OAd was determined by quantitative PCR. Ad neutralizing IgG in 1:1000 diluted serum was determined at the indicated time points. Neutralization was calculated on the basis of negative and positive controls.

On the basis of the preclinical data described above, we treated four patients with an ultralow dose (5 × 10^9^ vp) of CAdVEC (OAd:HDAd = 1:1) in our phase 1 clinical trial for patients with advanced solid tumors refractory to standard treatments (NCT03740256). This dosage of OAd is more than 100-fold lower than that used in other OAd clinical trials (*25*–*27*). Clinical details for patients are shown in Table 1. As expected, ULCA treatments were well tolerated with few and low-grade adverse events (Table 2), predominantly transient low-grade fever, and transient low-grade transaminitis (Fig. 4A). As in our preclinical murine studies, we observed increased levels of T_H_1-associated (IL-12p70 and IFN-γ) and T_H_2-associated (IL-10) cytokines at 4 days after ULCA (Fig. 4B). We also saw low-level elevation of TNF-α after ULCA treatment in all patients. All systemic increases in cytokines were transient, demonstrating that local ULCA treatment was well tolerated regardless of tumor site and type. We measured CAdVEC vector DNA copies in biopsies of the treated tumors 7 days after injection and detected both OAd and HDAd DNA in all tumor samples (Fig. 4C). Unlike other OAd trials, we did not detect circulating OAds in blood, urine, or buccal swab samples (Fig. 4C) (*25*–*27*), likely due to the low quantities injected. Despite the ULCA, all patients had increased titers of preexisting neutralizing immunoglobulin G (IgG) antibodies to Ad, consistent with the immunostimulatory properties of the study agent (Fig. 4C).

**Table 1.** Patients at baseline. SCC, squamous cell carcinoma.

Patient #	Dose	Age	Gender	Tumor type	Previous treatments
1	5 × 10^9^ vp	68	Female	Breast (ER/PR)	Tamoxifen, Taxotere, carboplatin, Faslodex, Ibrance, Xeloda, Kadcyla, olaparib
2	5 × 10^9^ vp	59	Female	Breast (ER/PR)	Avastin, Xeloda, carboplatin, paclitaxel, Cytoxan, Taxotere, everolimus, fulvestrant
3	5 × 10^9^ vp	67	Male	Oral SCC	Surgery, radiotherapy, and pembrolizumab
4	5 × 10^9^ vp	73	Female	Breast (ER/PR)	Donosumab, anastrozole, exemestane, Ibrance, Xgeva, tamoxifen, and everolimus

**Table 2.** Patient adverse events. AST, aspartate aminotransferase; ALT, alanine transaminase.

Event	Grade 1 events	Grade 2 events	Grade 3 events	Grade 4 events	Total events
Hematologic disorder					
Lymphocytopenia	0	1	1	0	2
Leukopenia	0	0	1	0	1
Neutropenia	0	0	1	0	1
Thrombocytopenia	1	0	0	0	1
General disorder or administration-site condition					
Injection site pain	0	1	0	0	1
Burning at injection site	1	0	0	0	1
Flu-like symptoms	2	0	0	0	2
Night sweats	1	0	0	0	1
Fever	1	0	0	0	1
Chills	2	0	0	0	2
Malaise	1	0	0	0	1
Fatigue	1	0	0	0	1
Nervous system disorder					
Headache	2	0	0	0	2
Myalgia	1	0	0	0	1
Gastrointestinal disorder					
Nausea	1	1	0	0	2
Vomiting	2	0	0	0	2
Elevated alkaline phosphatase	1	0	0	0	1
Elevated AST/ALT	2	0	0	0	2
Others					
Sinus tachycardia	1	0	0	0	1
Bone pain	1	0	0	0	1
				Total	27

### ULCA treatment modifies circulating immune cell profile and recruits CD8 T cells to tumor site

**Fig. 5.**
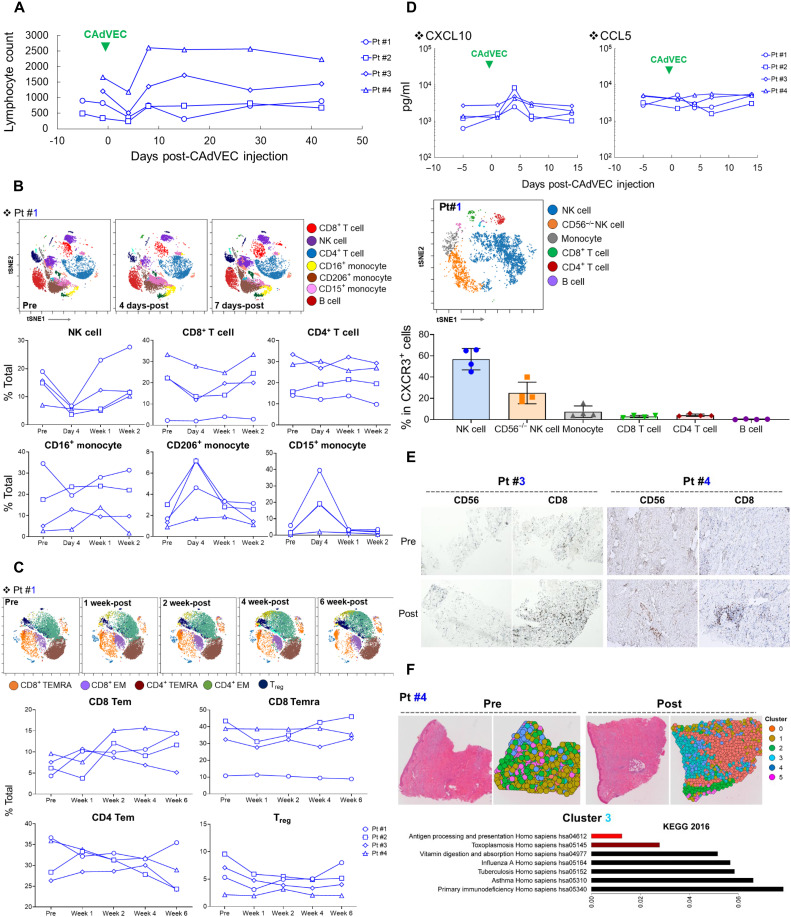
ULCA treatment changes circulating immune cell profile. (**A**) Lymphocyte numbers in PBMCs were counted at the indicated time points. (**B**) We phenotyped freshly isolated PBMCs with different immune cell markers (detailed in fig. S8) at the indicated time points. Flowsom data are representative data from patient #1. (**C**) We phenotyped freshly isolated PBMCs with different T cell markers (detailed in fig. S8) at the indicated time points. Flowsom data are representative data from patient #1. (**D**) We measured human CXCL10 and CCL5 levels in plasma samples by ELISA. We phenotyped freshly isolated CXCR3-positive PBMCs from patients before CAdVEC treatment (detailed in fig. S8). Flowsom data are representative data from patient #1. (**E**) Matched pre- and post-tumor biopsies from patients #3 and #4 were stained with human CD56 and CD8 IgG for immunohistochemistry (IHC). (**F**) We performed Visium spatial gene expression with pre- and post-CAdVEC tumor biopsy samples from patient #4. Immune cell signatures were performed using spaceranger count.

Because local OV treatment may induce lymphocytopenia and neutropenia (*28*, *29*), we monitored white blood cells in all patients on our trial. Lymphocyte counts only transiently decreased 4 days after ULCA injection in all patients (Fig. 5A and fig. S7). To better understand how ULCA initially affected lymphocyte subpopulations, we phenotyped peripheral immune cells and detected an early and transient decrease in both NK cells and CD8^+^ T cells; CD206^+^ and CD15^+^ monocytes, associated with immunosuppressive monocytes (*22*, *23*, *30*), were unchanged in total numbers but increased as a proportion of total peripheral blood mononuclear cells (PBMCs) in all patients (Fig. 5B and fig. S8A).

From 1 to 6 weeks after injection, we found that effector memory and terminally differentiated CD8^+^ T cells increased but not significantly in all patients (Fig. 5C and fig. S8B). In contrast to CD8^+^ T cells, we observed no longer-term changes in circulating effector memory CD4^+^ T cells. One week after ULCA, we saw a decrease in the number of circulating T cells with T_reg_-associated phenotype (CD4^+^, CD25^High^, CTLA-4^High^), and their levels remained below pretreatment values thereafter in all patients. These results are consistent with skewing of local and systemic immune responses toward an actively cytotoxic pattern.

Previous humanized mouse studies indicated that intratumoral injection of CAdVEC induced expression of chemokines CXCL10 and CCL5 at tumor sites (*15*). We found that CXCL10, but not CCL5, was transiently increased in the plasma of all patients (Fig. 5C). Consistent with their appearance at the tumor site, we found that NK cells in patient peripheral blood most consistently expressed CXCR3, the receptor for CXCL10 (Fig. 5, D and E, and fig. S8C). The CXCR3/CXCL (CXCL9 to CXCL11) axis is an important role for NK cell trafficking to virus-infected site (*31*). Although CD8^+^ T cells are minor populations in CXCR3^+^ PBMCs, ULCA treatment also increased the numbers of CD8^+^ T cells at the tumor site (Fig. 5E). While CXCR3 on CD8^+^ T cells is important for recruitment to virus-infected sites (*31*), CCR5 expression on CD8^+^ T cells is required for the localization of CD8^+^ T cells at the infected site (*32*), suggesting that other chemokines or chemokine receptors contribute to this T cell localization at the tumor site. These findings suggest that local ULCA treatment–dependent chemotaxis recruits CD8 T cells to the tumor site. To understand whether this recruitment altered the TME gene signature, we performed Visium spatial gene expression profiling on pre- and post-CAdVEC tumor samples from patient #4 (Fig. 5F). We generated spatial plots with the Seurat R package using the tools for normalization, dimensional reduction, clustering, and detecting spatially variable features (*33*). Each spot was clustered on the basis of spatial gene expression analysis. Genes were sparsely distributed in both pre- and post-CAdVEC tumor samples; however, the mean number of genes per spot increased in the post-CAdVEC sample. To compare the samples, we merged the data and performed spatially resolved differential gene expression analysis between spots, generating a list of genes with increased expression after CAdVEC that we could compare with the Kyoto Encyclopedia of Genes and Genomes (KEGG) pathway database. Gene set enrichment analysis of clusters before and after treatment showed significant enrichment of the “Antigen Processing and Presentation” pathway (cluster 3) following ULCA. These results suggest that chemotaxis induced by local ULCA treatment repolarizes the TME toward an immunologically hot environment, replicating our observations in humanized mice.

### ULCA stimulates antitumor activity in patients

**Fig. 6.**
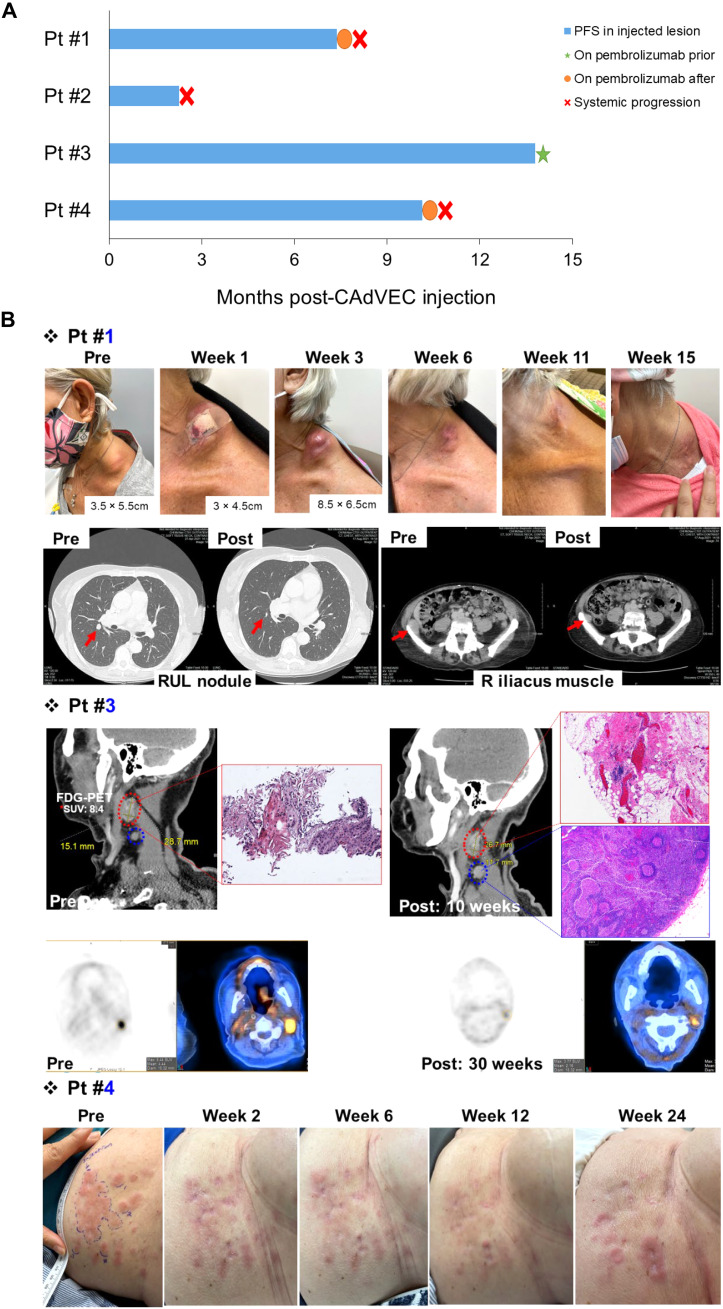
ULCA treatment stimulates antitumor activity. (**A**) Swimmers plot of CAdVEC injected tumor in each patient after ULCA treatment. (**B**) Each patient’s tumor images at the indicated time points. Some patients also had scans at the indicated time points.

Three of four patients treated with a single ULCA showed tumor responses, including one surgically confirmed complete response (CR) (Fig. 6, A and B). Following the initial 6-week evaluation of toxicity and efficacy, all patients subsequently received pembrolizumab (anti–PD-1 IgG) after ULCA treatment (fig. S9).

Patient #1 had metastatic ER/PR^+^ breast cancer and had failed multiple standard systemic therapies (Table 1). We injected ULCA into a metastatic left cervical neck nodule. The patient developed progression of disease in distant brain metastases and was started on pembrolizumab 3 weeks after ULCA injection. The ULCA-injected tumor decreased at week 6 after injection and became barely palpable on exam at week 15. A computed tomography (CT) scan at week 15 confirmed overall partial response by RECIST 1.1 with responses in a distant lung and the iliacus muscle metastases.

Patient #2 had metastatic ER/PR^+^ breast cancer and had failed multiple standard systemic therapies (Table 1). We injected ULCA into a metastatic liver tumor. A CT scan at week 10 showed progressive disease based on RECIST 1.1 both at the site of the injection and with other hepatic lesions. The patient was started on pembrolizumab 10 weeks after ULCA injection, following the staging scan but continued to progress.

Patient #3 had Human Papillomavirus (HPV)-negative recurrent head and neck squamous cell carcinoma after surgery, radiation, and chemotherapy. He was on pembrolizumab before his ULCA injection into a metastatic left cervical neck nodule (Table 1 and fig. S7). At week 10 after injection, there was radiographic concern for progression with new locoregional lymphadenopathy. After multidisciplinary tumor board recommendations, a left neck dissection was performed at week 14. All resected tissues showed a complete pathologic response, and a subsequent positron emission tomography (PET)–CT scan at week 28 confirmed a CR based on RECIST 1.1 with no residual or distant disease. He remains disease free without further treatment at month 15 after injection.

Patient #4 had metastatic, recurrent ER/PR^+^ breast cancer (Table 1) with measurable cutaneous metastases. A single measurable cutaneous metastasis of her abdomen was injected with ULCA. Both the injected and surrounding cutaneous tumors showed partial response (PR) 2 weeks after injection. At week 6, the patient had PR based on RECIST 1.1. Her skin lesions continued to improve with a sustained PR measured by RECIST criteria up to 24 weeks after injection. She had no visceral metastasis on all staging scans up to 24 weeks.

## DISCUSSION

Despite encouraging preclinical studies, OAds have had limited success in patients with advanced solid tumors, suggesting that preclinical doses relative to the tumor size did not correspond to human disease. Substantive increases in OAd dosage are limited by technical constraints and systemic toxicities. Here, we demonstrated that local ULCA treatment can control tumor growth through oncolysis and stimulation of host antitumor responses in both preclinical and clinical studies.

Immunostimulatory molecules from Armed OAds are diminished after lysis of OAd-infected cells by Ad-specific T cells. Immunostimulatory molecules in CAdVEC, however, are expressed by HDAd, which has the same infectivity as OAd but cannot replicate and does not express adenoviral genes in infected cells, leading to constitutive transgene expression both in malignant cells spared from OAd lysis and in the supporting tumor stroma (*5*). Because OAds generally have limited infectivity and replication in rodent cell lines (*34*, *35*), we tested whether these independent but additive antitumor mechanisms (OAd: tumor lysis, HDAd: immune stimulation) enable control of tumor growth with ULCA in breast cancer xenograft (oncolysis) and humanized (immune stimulation) mouse models. We found that ULCA significantly controlled breast tumor growth in xenograft mice even at doses 1000-fold lower than other OAd xenograft mouse studies (*10*–*12*).

Humanized mice with MCF-7 tumors develop metastases similar to those seen in xenograft mice, but treatment with ULCA not only controlled MCF-7 primary tumor growth but also prevented metastasis in this model. Furthermore, ULCA was sufficient to resolve tumors in both the MCF-7 (immunologically cold) and SUM-159 (hot tumors with higher baseline immune cell infiltrate) humanized mice. Although we do not yet know the full range of tumors for which this therapy will work, these findings demonstrate that CAdVEC elicits potent antitumor activity regardless of background immune cell infiltration in breast cancer models.

In humanized mice, we found that local ULCA treatment leads to T_H_1 and T_H_2 cytokine circulation in the blood, similar to our subsequent observations in patients. Despite the increase in cytokines, we saw no treatment-related toxicities (weight loss and cytokine release syndrome) in our mice or patients. This safety profile was anticipated given that ULCA dosage was 1000-fold lower than other OAd preclinical studies (*10*–*12*) and 100-fold lower than other OAd clinical trials (*25*–*27*). In addition to being safe, ULCA locally repolarized the TME toward an immunologically hot environment, increasing CD8^+^ T cell and NK cell infiltration in both humanized mice and patients. Although we found increased CD8^+^ T cell infiltration in CAdVEC-injected tumors of patients, we could not further analyze them (e.g., single-cell RNA sequencing) because of limited sample availability. Because increases in activated CD8^+^ T cell infiltration correlated with adaptive immune responses against cancer cells in humanized mice, and we can generate more than 100 humanized mice from a single cord blood unit (CBU; minimizing immunological heterogeneity seen among patients), deeper analysis of humanized mouse models may afford accurate prediction of local and systemic immune responses following local CAdVEC treatment.

In four patients treated with a single ULCA expressing IL-12p70, PD-L1 antibody, and HSVtk safety switch, we saw one CR and two PRs in injected lesions, as well as abscopal effects in untreated metastatic tumors. Additional ICI treatment after ULCA injection led to prolonged responses, including control of ULCA-untreated tumor growth in patient #1. Although head and neck cancer (HNC) is an immunologically hot tumor and ICI treatment is approved by the U.S. Food and Drug Administration (FDA) (*36*), our HNC patient progressed on ICI before ULCA injection. In addition, checkpoint blockade generally had modest effects in ER/PR^+^ patients in phase 1 clinical trials (*37*). Because we demonstrated that local CAdVEC injection led to IFN-γ induction in both preclinical (humanized mice) and clinical studies, and that IFN-γ induces PD-L1 expression on cancer cells (*6*), it is likely that tumor cells per site in human subjects also express PD-L1 after CAdVEC injection. If so, then CAdVEC-derived PD-L1 blocker should locally inhibit PD-1:PD-L1 interaction with enhanced local antitumor activity via stimulation of immune effector cells (*38*). However, because CAdVEC-derived PD-L1 blocker produces minimal circulating levels of the agent, the combination of local PD-L1 blocker followed by systemic administration should augment the initial antitumor activity within the CAdVEC-treated tumor site and support extension and continuation of this response systemically. As patient numbers increase and more detailed analysis of the temporal sequence of events locally and abscopally becomes feasible, it will be possible to determine with greater certainty whether the benefits of sequential combination of low-dose CAdVEC and exogenous immune checkpoint inhibition are complementary in an analogous way to the combination of talimogene laherparepvec (T-VEC) and ICI in melanoma patients (*39*).

This highly potent CAdVEC treatment is easy to administer and appears safe at a dose that is nonetheless sufficient to produce significant clinical responses. While a single ULCA treatment was sufficient to resolve some tumors, not all patients achieved CRs. Because HDAd has a large cargo capacity (up to 34 kb), and our current HDAd uses only 11 kb (IL-12p70, PD-L1 antibody, and HSVtk safety switch), incorporation of additional immunostimulatory transgenes is certainly feasible and may also be desirable to further enhance host antitumor activity.

## MATERIALS AND METHODS

### Study design

This research was approved by the Institutional Review Board. The overall objective of this study was to evaluate the therapeutic potential of ULCA expressing IL-12p70, anti–PD-L1 antibody, and HSVtk safety switch in preclinical and clinical studies. We evaluated the antitumor efficacy, TME repolarization, and development of adaptive immune responses to ULCA treatment in breast cancer xenograft and humanized mouse models. In these preclinical studies, animals were randomly allocated to experimental groups with similar mean tumor volumes and mean body weights, but experiments were not blinded.

We treated ULCA to four adult patients with advanced solid tumors at Baylor College of Medicine (NCT03740256). Treatment safety and antitumor efficacy, including local and systemic immune responses, were evaluated.

### Cell lines and adenoviral vectors (HDAds and OAds)

We obtained human breast lines MCF-7 and SUM-159 and murine breast line 4T1 from the American Type Culture Collection (ATCC; Manassas, VA) in 2018. We authenticated cell lines with short tandem repeat profiling by ATCC. Cells were cultured under the conditions recommended.

To generate cell lines expressing the fusion protein enhanced green fluorescent protein (EGFP)–*ffLuc*, we infected cells with retrovirus encoding EGFP-*ffLuc* (*7*, *40*). EGFP-positive cells were sorted using an SH800 cell sorter (Sony) after three passages after infection of retrovirus. The human adenoviral vectors OAd5/3Ad2E1AΔ24 and HDAd*Trio* were previously developed and characterized in detail (*15*, *41*).

### Animal experiments

The Baylor College of Medicine Institutional Animal Care and Use Committee approved all animal experiments. For the orthotopic xenograft models, 2 × 10^6^ MCF-7 cells or 2 × 10^6^ SUM-159 cells expressing *ffLuc* were resuspended in a volume of 100 μl of Matrigel and injected into the mammary fat pad of 7- to 8-week-old NSG female mice (SUM-159) and NSG*SGM3* female mice (MCF-7). After tumor volumes reached >100 mm^3^, a total of 1 × 10^6^ vp of CAdVEC (OAd:HDAd = 1:1) were injected in a volume of 20 μl into the primary tumor. The ratio of OAd to HDAd in the CAdVEC system was optimized to effectively propagate transgene(s) encoded in the coinjected HDAd with lytic effects even with clinically relevant dosages (IND19439). Control mice received vehicle [phosphate-buffered saline (PBS)] alone. Cancer cells expressing *ffluc* were assessed using the In Vivo Imaging System (Xenogen) (*42*). The end point was established at a tumor volume of >1500 mm^3^ or a body weight of <80%.

For the humanized mouse model, we crossed NSG*SGM3* with NSG*HLA-A2* (NSG*A2_SGM3*). We then humanized these transgenic mice with HLA-A2^+^CD34^+^ cells (fig. S2A). Newborn (1 to 2 days from birth) female NSGA2_SGM3 (crossed with NSGTG^CMV-IL3,CSF2,KITLG^ Eav/mloySz and NSGTG^HLA-A/H2-D/B2M^ Dvs/SzJ: The Jackson Laboratory) were sublethally irradiated (100 cGy) and intrahepatically injected with 5 × 10^4^ human CBU-derived CD34^+^ cells. CBUs were obtained from MD Anderson Stem Cell Center. CD34^+^ cells were isolated using a CD34^+^ cell isolation kit (Miltenyi Biotec Inc.) and expanded with StemSpan CD34^+^ Expansion Supplementation for 3 days (STEMCELL Technologies). After confirming human CD45^+^ cells in PBMCs of mice 8 to 9 weeks after injection, 2 × 10^6^ MCF-7 or SUM-159 cells expressing *ffLuc* were resuspended in a volume of 100 μl of Matrigel and injected into the mammary fat pad. After tumor volumes reached >100 mm^3^, a total of 1 × 10^6^ vp of CAdVEC (OAd:HDAd = 1:1) were injected in a volume of 20 μl into the primary tumor. Control mice received vehicle (PBS) alone. Cancer cells expressing *ffluc* were assessed using the In Vivo Imaging System (*42*). The end point was established at a tumor volume of >1500 mm^3^ or a body weight of <80%.

### Isolation of tumor-infiltrating immune cells

After rinsing harvested tumors with PBS, tumors were minced and incubated in RPMI medium containing human tumor dissociation reagents (Miltenyi Biotec Inc.) at 37°C for 1 hour. Cells were passed through a 70-μm cell strainer (BD Pharmingen), and murine stroma cells were removed using a Mouse Cell Depletion kit (Miltenyi Biotec Inc.). Human cells were stained with the antibodies described in Results and the figure legends.

### Flow cytometry

We used the following fluorochrome-conjugated monoclonal antibodies: anti-human CD3, CD4, CD8, CD25, TIM-3, CD278, LAG-3, CTLA-4, CD134, CD137, CCR7, CD45RO, PD-1, PD-L1, CD20, CD56, CD14, CD33, CD11b, CD11c, CD16, CD80, CD206, and CD15 (BD Biosciences, Beckman Coulter, BioLegend). Cells were stained with these antibodies or the appropriate isotype control antibodies for 30 min at 4°C. We determined live/dead discrimination via exclusion of Fixable Viability Stain 780–positive cells (BD Biosciences). Stained cells were analyzed using BD Symphony (BD Biosciences) at the Flow Cytometry Core at Texas Children’s Hospital. We analyzed the data with Kaluza software (Beckman Coulter) and Cytobank (Cytobank Inc.) according to the manufacturer’s instructions.

### IFN-γ ELISPOT assay

Splenocytes were harvested from humanized mice at 24 days after CAdVEC injection, and human CD8^+^ T cells were isolated using CD8 isolation column (Miltenyi Biotec Inc.). The day before CD8^+^ T cell isolation, parental MCF-7 and SUM-159 cells were seeded in the presence of human IFN-γ (10 ng/ml), and single-cell suspensions were subjected to 100 Gy of irradiation the next day. CD8^+^ T cells (4 × 10^5^) were mixed with 4 × 10^4^ irradiated cancer cells (effector:target = 10:1) in 200 μl of complete medium and seeded in a 96-well Millipore ELISPOT plate (BD Biosciences) that was precoated with IFN-γ capture antibody (BD Biosciences) (*24*). Plates were cultured for 24 hours in a 37°C incubator and then developed according to the manufacturer’s protocol. Plates were scanned using Mabtech IRIS Elispot reader (Mabtech).

### Clinical trial

CAdVEC used for the clinical study was identical to the combination agent used for the preclinical investigation, containing an OAd and an HDAd encoding IL-12p70, anti–PD-L1 antibody, and HSVtk safety switch. The four patients with advanced metastatic tumors refractory to conventional therapies, including ICIs, were treated with a single injection of CAdVEC at Baylor College of Medicine (NCT03740256) (Table 1). All had Eastern Cooperative Oncology Group (ECOG) performance of 0 to 2 and no major organ function deficiencies. Other exclusion criteria were organ transplant, HIV, severe cardiovascular, and metabolic or pulmonary disease (e.g., symptomatic coronary heart disease and uncontrolled blood pressure).

The primary end point for this study was incidence of dose-limiting toxicities (DLTs) after a 6-week evaluation period. Secondary outcomes included overall response rates, disease control rate, progression free survival (PFS), overall survival, and the number of treatment-related adverse events. Study entry required written informed consent, and the study itself followed Good Clinical Practice guidelines under an approved FDA Investigational New Drug (IND) application.

Patients received an image-guided intratumoral injection of CAdVEC and then monitored overnight in the hospital and followed for a 6-week DLT evaluation period. Adverse events were graded according to CTCAE v5.0. Response Evaluation Criteria in Solid Tumors v1.1 (RECIST) was applied to evaluate disease response, including injected and untreated lesions. These criteria include CR, PR (>30% reduction in the sum of tumor diameters), stable disease (i.e., no response/progression), and progressive disease (>20% increase).

### Quantification of vector genome DNA in CAdVEC-injected tumors of patients

Total DNA was extracted from 7 days after tumor biopsy, urine, buccal swab, and serum samples with a DNeasy Blood and Tissue kit (QIAGEN), and DNA concentrations were measured using NanoDrop 2000. Vector copies were quantified with primer sets for OAd [5′-TCCGGTTTCTATGCCAAACCT-3′, 5′-TCCTCCGGTGATAATGACAAGA-3′, and FAM 5′-TGATCGATCCACCCAGTGA-3′ (probe)] and HDAd (5′-TCTGAATAATTTTGTGTTACTCATAGCGCG-3′, 5′-CCCATAAGCTCCTTTTAACTTGTTAAAGTC-3′, and FAM 5′-TGACCGTTTACGTGGAGACTCGCCCA-3′ (probe)]. On the basis of standard curve with original plasmid DNA, vector copies were calculated per microgram.

### Immunohistochemistry

The Human Tissue Acquisition and Pathology Core at Baylor College of Medicine stained tumor biopsies from patients with anti-human CD8 antibody.

### Cytokine analysis

We collected blood samples from preclinical studies (xenograft and humanized mouse models) and clinical studies at the time points indicated in Results and the figure legends. We ran Multiplex Cytokine Immunoassay (EMD Millipore) from these plasma samples.

### Ad neutralizing IgG analysis

We measured Ad neutralizing IgG levels in the blood of preclinical (humanized mouse model) and clinical samples with modified previous protocols. Briefly, heat-inactivated (56°C, 30 min) plasma and serum samples were diluted to 1:10, 1:100, and 1:1000. HDAd5/3EGFP was mixed with diluted serum (plasma) samples and incubated at 37°C for 1 hour. A549 cells in duplicates were infected with 1000 vp/cell in 50 μl of diluted serum and incubated at 37°C for 30 min. Cells were also infected with serially diluted HDAd5/3EGFP (1000 to 10 vp/cell) for standard curve. We washed cells with PBS once and added medium at 150 μl/well. After 24 hours, we measured EGFP positivity with IncuCyte. To evaluate neutralizing antibodies in serum (plasma) samples, we plotted EGFP positivity relative to serially diluted HDAd5/3EGFP and determined neutralizing antibody activities.

### 10X Visium analysis

We prepared sections from pre- and post-CAdVEC Formalin-Fixed Paraffin-Embedded (FFPE) from patient #4. Total RNA was extracted from sections using the RNeasy Plus Mini Kit and quantified using NanoDrop 2000. The Baylor Genomic & RNA Profiling and Histopathology Cores performed RNA expression profiling with the Visium Spatial Gene Expression (10X Genomics). Data quality control, normalization, and advanced analysis were performed using spaceranger count (1.3.0) following Visium Spatial Gene Expression guidelines. Spatial plots were generated with the Seurat R package for spatial gene expression analysis (4.1.0), using the function SpatialDimPlot after normalization, and dimensional reduction was performed with Uniform Manifold Approximation and Projection (UMAP) (*33*). Differentially expressed genes were identified with the Wilcoxon rank sum test, using the Seurat function FindMarkers with default parameters.

### Statistics and reproducibility

Results are represented as means of two or more independent experiments (biological replicates). Data with three or more groups were analyzed by ordinary one-way analysis of variance (ANOVA). Wilcoxon matched pairs test was used to compare two groups of paired data. Data were analyzed with GraphPad Prism 9.
