# Supplementary material for: Ultralow-dose binary oncolytic/helper-dependent adenovirus promotes antitumor activity in preclinical and clinical studies
Source: Sci Adv. 2023 Mar 29;9(13):eade6790. doi: 10.1126/sciadv.ade6790 (PMC10058234; doi:10.1126/sciadv.ade6790)
Supplement: Supplementary file 1 — Figs. S1 to S9 [file sciadv.ade6790_sm.pdf]

Science Advances
AAAS

Supplementary Materials for

Ultralow-dose binary oncolytic/helper-dependent adenovirus promotes antitumor activity in preclinical and clinical studies

Daniel Wang *et al.*

Corresponding author: Masataka Suzuki, suzuki@bcm.edu

Sci. Adv. **9**, eade6790 (2023)
DOI: 10.1126/sciadv.ade6790

This PDF file includes:

Figs. S1 to S9

Supplementary Materials

Supplementary figures

Figure S1

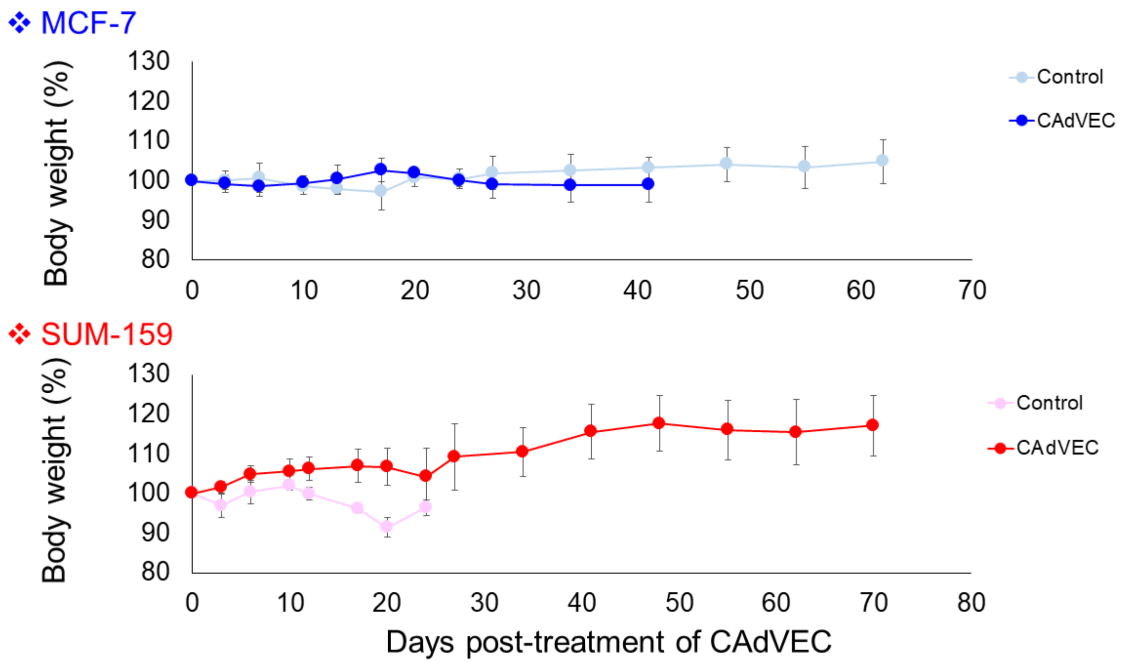

Fig. S1. CAdVEC treatment does not cause weight loss in breast cancer xenograft models. ffLuc-labeled MCF-7 and SUM-159 cells were orthotopically transplanted into the mammary fat pad of NSG female mice (n=5 animals per condition). After tumor volume reached > 100mm^3, we injected a total of 1x10^6 vp of CAdVEC (OAd:HD=1:1) intratumorally. Control mice received vehicle (PBS) alone. We monitored animal body weights at the indicated time points. Data are presented as means ± SD.

Figure S2

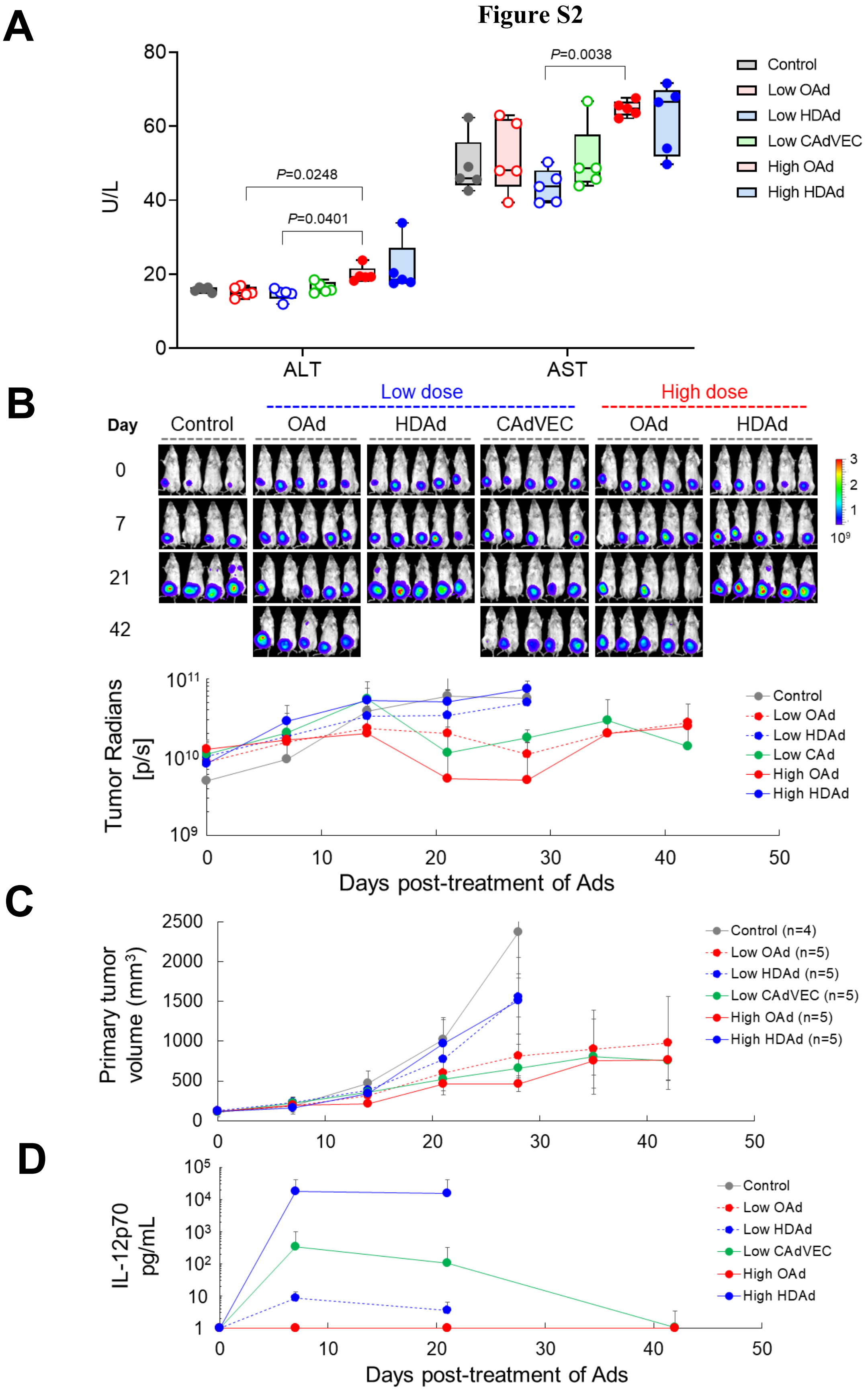
A
U/L
P=0.0248
P=0.0401
P=0.0038
ALT
AST
Control
Low OAd
Low HDAd
Low CAdVEC
High OAd
High HDAd
B
Low dose
High dose
Day
Control
OAd
HDAd
CAdVEC
OAd
HDAd
0
7
21
42
Tumor Radians [p/s]
Days post-treatment of Ads
Control
Low OAd
Low HDAd
Low CAd
High OAd
High HDAd
C
Primary tumor volume (mm3)
Control (n=4)
Low OAd (n=5)
Low HDAd (n=5)
Low CAdVEC (n=5)
High OAd (n=5)
High HDAd (n=5)
D
IL-12p70 pg/mL
Days post-treatment of Ads
Control
Low OAd
Low HDAd
Low CAdVEC
High OAd
High HDAd

Fig. S2. **Single agents show dose-dependent anti-tumor effects and toxicity in SUM-159 orthotopic xenograft model**. **(A)** ffLuc-labeled SUM-159 cells were orthotopically transplanted into the mammary fat pad of NSG female mice (n=5 animals per condition). After tumor volume reached > 100mm^3, we injected a total of 1x10^6 vp of OAd, HDAd, CAdVEC (OAd:HD=1:1), 1x10^9 vp of OAd, HDAd intratumorally. Control mice received vehicle (PBS) alone. We measured liver enzymes AST and ALT at 3 days post-injection. **(B)** We monitored tumor bioluminescence at the indicated time points. Data are presented as means ± SD. **(C)** Primary tumor volumes were monitored at different time points. **(D)** We collected serum samples from mice at 0, 3, 7, 21 and 42 days post-injection of CAdVEC, and measured IL-12p70 levels by ELISA. Data are presented as means ± SD.

Figure S3

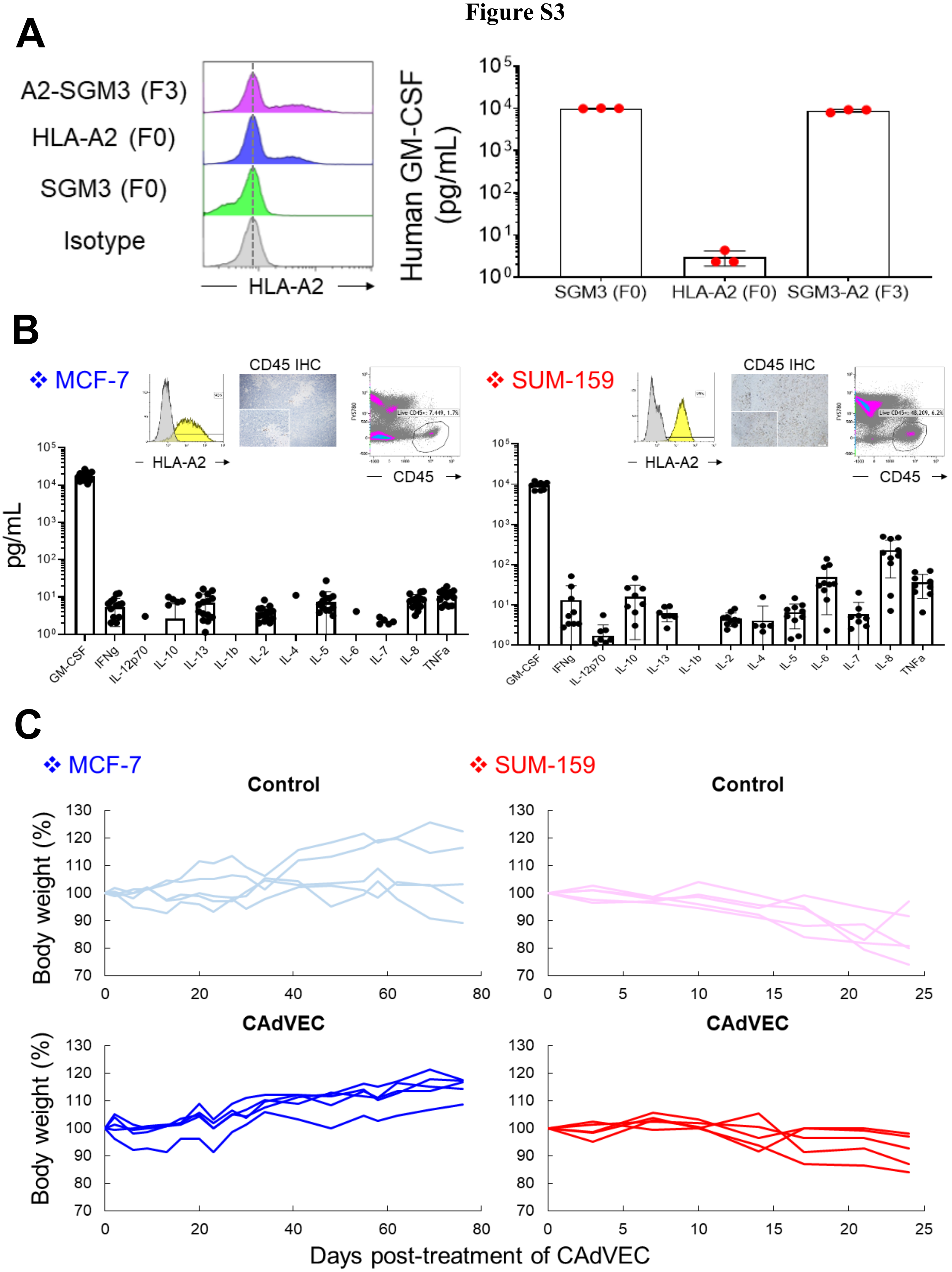
A
A2-SGM3 (F3)
HLA-A2 (F0)
SGM3 (F0)
Isotype
HLA-A2
Human GM-CSF (pg/mL)
SGM3 (F0)
HLA-A2 (F0)
SGM3-A2 (F3)
B
MCF-7
CD45 IHC
HLA-A2
CD45
SUM-159
pg/mL
GM-CSF
IFNg
IL-12p70
IL-10
IL-13
IL-1b
IL-2
IL-4
IL-5
IL-6
IL-7
IL-8
TNFa
C
MCF-7
SUM-159
Control
CAdVEC
Body weight (%)
Days post-treatment of CAdVEC

Fig. S3. **Humanized transgenic NSG*SGM3_HLAA2* mice form tumors after transplantation of HLA-partially matched breast cancer cells**. **(A)** We crossed NSGSGM3 and NSGHLA-A2. After confirming homozygous mice (F3) by genotyping, we phenotyped human HLA-A2 expression on circulating murine cells and measured human GM-CSF levels in the blood of these transgenic mice. **(B)** We orthotopically transplanted HLA-A2+ MCF-7 and SUM-159 cells into the mammary fat pad of humanized female mice. After tumor volume reached > 100mm^3, we collected tumors and performed human CD45 IHC and flowcytometry. We also collected serum samples and measured human Th1 and Th2 cytokine levels by Multiplex. Data are presented as means ± SD. **(C)** ffLuc-labeled MCF-7 and SUM-159 cells were orthotopically transplanted into the mammary fat pad of humanized female mice (n=5 animals per condition). After tumor volume reached > 100mm^3, we injected a total of 1x10^6 vp of CAdVEC (OAd:HD=1:1) intratumorally. Control mice received vehicle (PBS) alone. We monitored animal body weights at different time points.

Figure S4

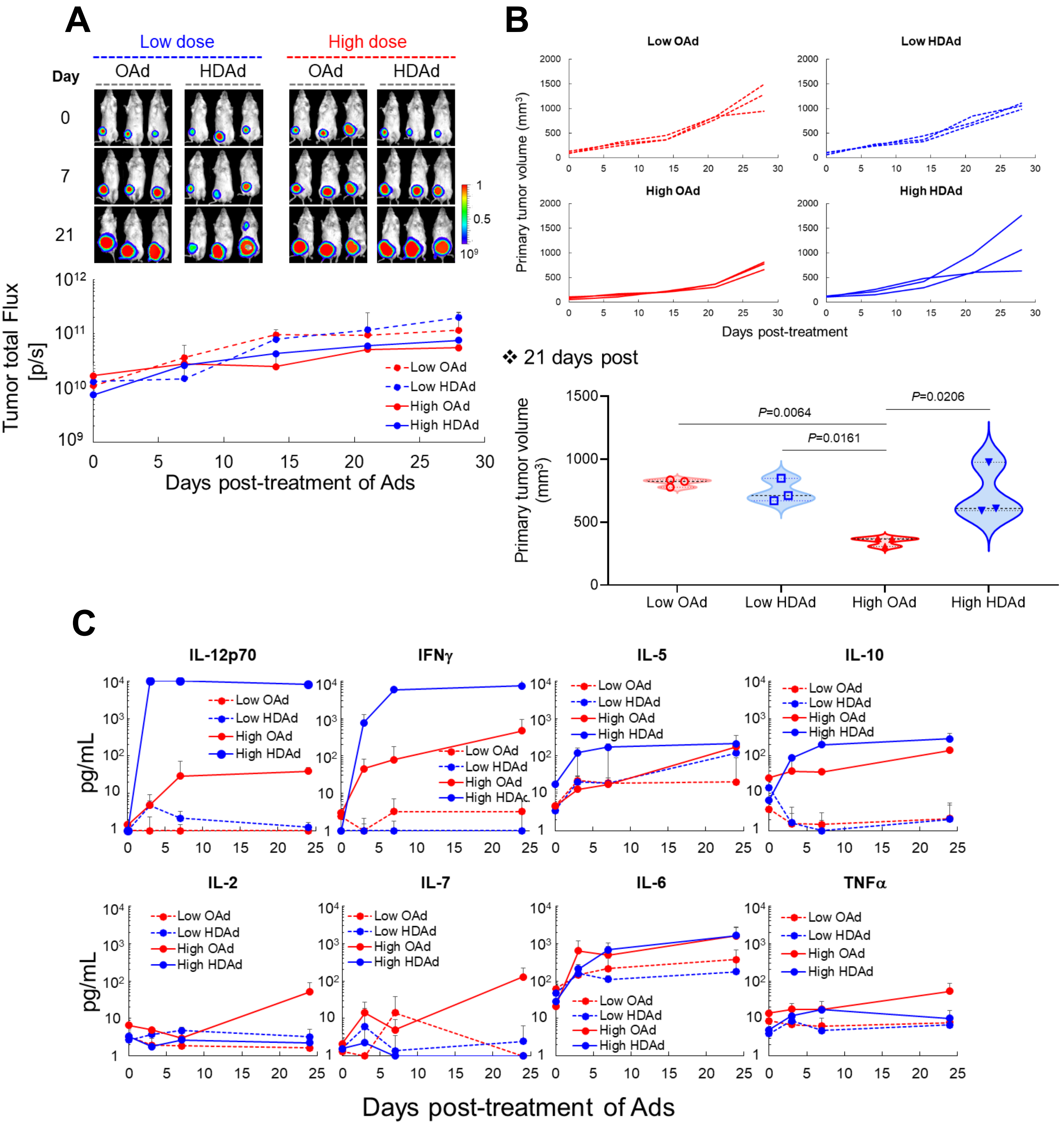

A
Low dose
High dose
Day
OAd
HDAd
OAd
HDAd
0
7
21
1
0.5
10^9
Tumor total Flux [p/s]
10^{12}
10^{11}
10^{10}
10^9
Low OAd
Low HDAd
High OAd
High HDAd
Days post-treatment of Ads
B
Low OAd
Low HDAd
High OAd
High HDAd
Primary tumor volume (mm^3)
Days post-treatment
❖ 21 days post
Primary tumor volume (mm^3)
P=0.0064
P=0.0161
P=0.0206
Low OAd
Low HDAd
High OAd
High HDAd
C
IL-12p70
IFNγ
IL-5
IL-10
IL-2
IL-7
IL-6
TNFα
pg/mL
Low OAd
Low HDAd
High OAd
High HDAd
Days post-treatment of Ads

Fig. S4. **Single agents show dose-dependent inflammation in SUM-159 orthotopic humanized mouse model**. **(A)** ffLuc-labeled SUM-159 cells were orthotopically transplanted into the mammary fat pad of humanized female mice (n=3 animals per condition). After tumor volume reached > 100mm^3, we injected i.t. a total of 1x10^6 vp of OAd, HDAd, 1x10^9 vp of OAd or HDAd. We monitored tumor bioluminescence at the indicated time points. Data are presented as means ± SD. **(B)** Primary tumor volumes were monitored at different time points. **(C)** We collected serum samples from mice at 0, 3, 7 and 24 days post-injection of Ad vectors, and measured human Th1 and Th2 cytokine levels by Multiplex. Data are presented as means ± SD.

Figure S5

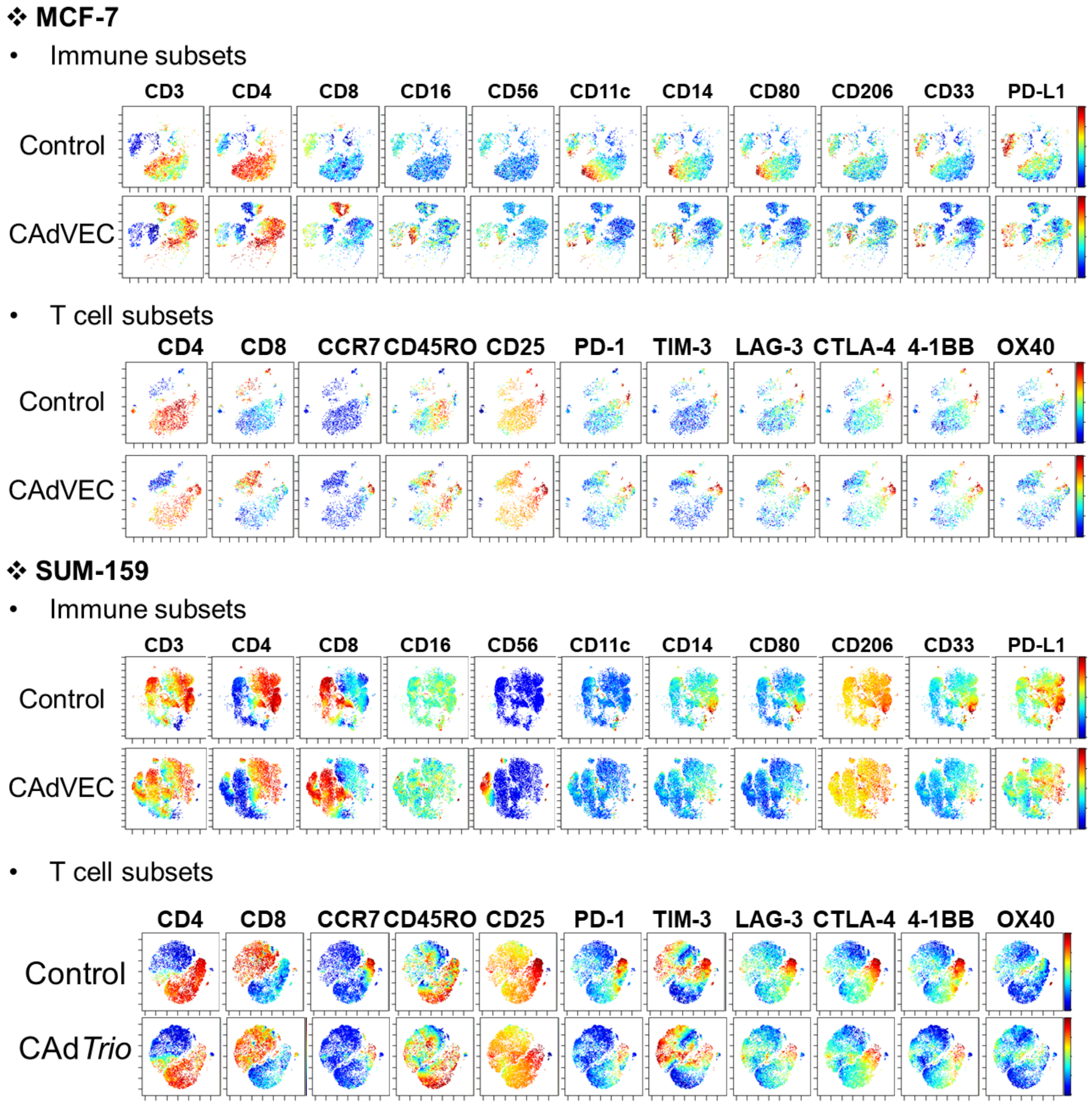

Fig. S5. **tSNE plots of single human marker staining in humanized mice**. MCF-7 and SUM-159 cells were orthotopically transplanted into the mammary fat pad of humanized female mice. After tumor volume reached > 100mm^3, a total of 1x10^6 vp of CAdVEC (OAd:HD=1:1) were injected i.t.. Tumor samples were collected at 24 days post-CAdVEC injection. Immune cells were stained with indicated markers and plotted on tSNE.

Figure S6

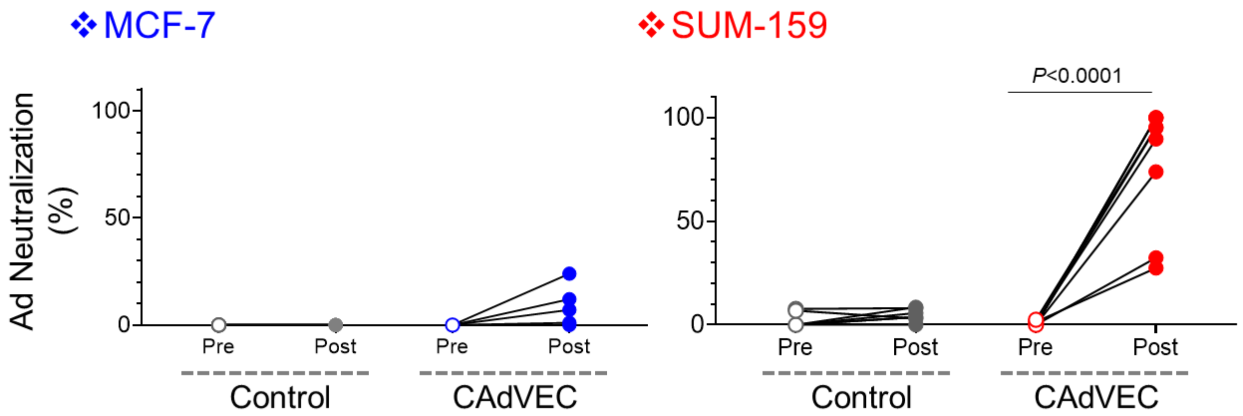

Fig. S6. **Ad neutralizing IgG development in humanized mice**. MCF-7 and SUM-159 cells were orthotopically transplanted into the mammary fat pad of humanized female mice. After tumor volume reached > $100mm^3$, a total of $1x10^6$ vp of CAdVEC (OAd:HD=1:1) were injected i.t.. Blood samples were collected at 24 days post-CAdVEC injection, Ad neutralizing antibody in 1/10 diluted serum was analyzed. Neutralization were calculated based on negative and positive controls. The Ad neutralizing antibody assay is described in **Materials and Methods**.

Figure S7

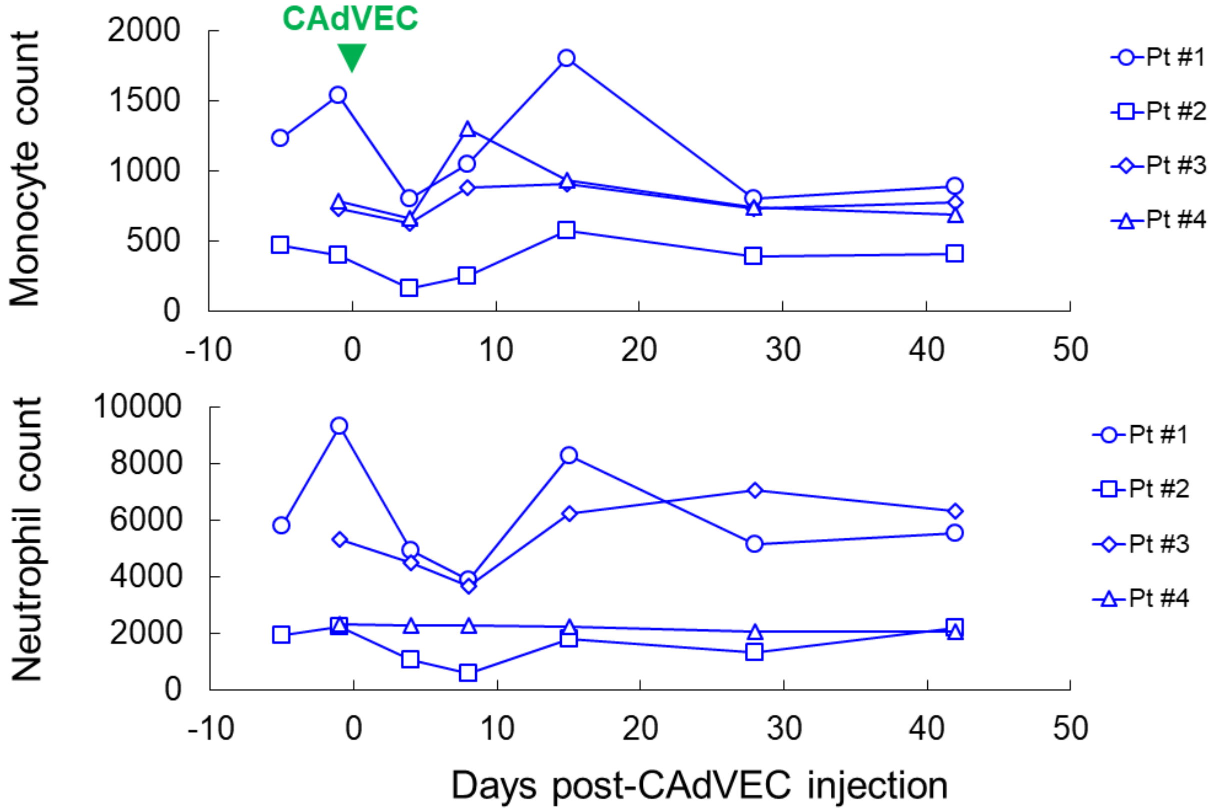

Fig. S7. **Peripheral monocyte and neutrophil counts in patients**. A total of $5x10^9$ vp of CAdVEC (OAd:HD=1:1) in 500 μL were intratumorally injected. Monocyte and Neutrophil numbers in peripheral blood were counted at the indicated time points.

Figure S8

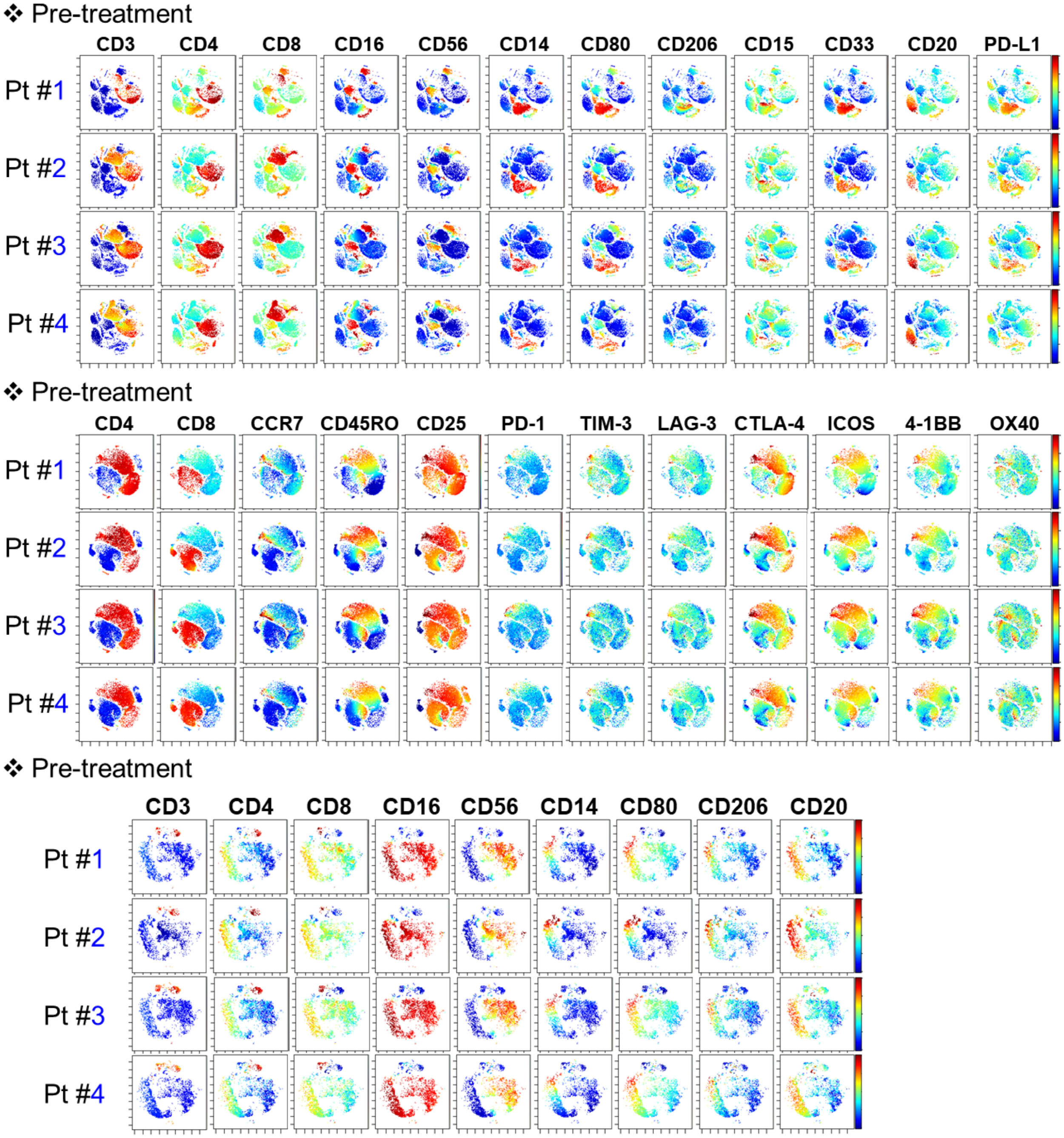

Fig. S8. **tSNE plots of single human immune cell marker staining in patient PBMCs**. A total of $5x10^9$ vp of CAdVEC (OAd:HD=1:1) in 500 μL were intratumorally injected. We phenotyped freshly isolated PBMCs with different immune cell markers and plotted on tSNE.

Figure S9

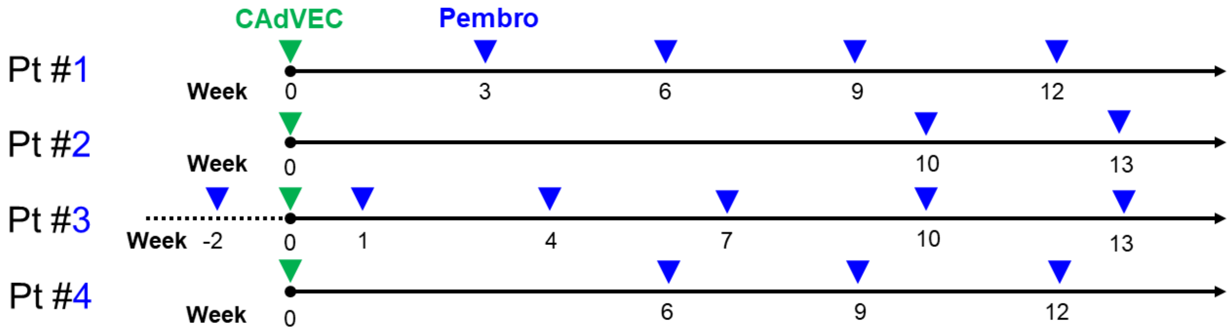

Fig. S9. **Checkpoint inhibitor (Pembrolizumab) treatment calendar in patients**. Patients were treated with Pembrolizumab at the indicated time points.
